# Pulmonary Function in Parkinson’s Disease: A Comparative Study of Spirometry and Impulse Oscillometry

**DOI:** 10.3390/biomedicines14051176

**Published:** 2026-05-21

**Authors:** Alexandra-Cristiana Gache, Elena Danteș, Ariadna-Petronela Fildan, Andreea-Cristina Postu, Viorica Zamfir, Adina-Milena Man, Nicoleta-Larisa Șerban, Irene Rășanu, Any Axelerad

**Affiliations:** 1 Department of Pneumology, Faculty of Medicine, Campus—Corp B, Ovidius University of Constanta, 1 University Alley, 900470 Constanta, Romania; alexandra.belu@365.univ-ovidius.ro (A.-C.G.); elena.dantes@365.univ-ovidius.ro (E.D.); petronela.fildan@365.univ-ovidius.ro (A.-P.F.); viorica.zamfir@365.univ-ovidius.ro (V.Z.); 2 Medical Doctoral School, Faculty of Medicine, Campus—Corp B, Ovidius University of Constanta, 1 University Alley, 900470 Constanta, Romania; irenedamian@yahoo.com (I.R.); docuaxi@yahoo.com (A.A.); 3 Clinical Hospital of Pneumopthisiology Constanta, 40 Santinelei Street, 900002 Constanta, Romania; 4 “St. Ap. Andrew” Emergency County Clinical Hospital, 145 Tomis Boulevard, 900591 Constanta, Romania; 5 Department of Pneumology, “Iuliu Hatieganu” University of Medicine and Pharmacy, 400012 Cluj-Napoca, Romania; manmilena50@yahoo.com; 6 Department of Neurosurgery, Cluj County Clinical Emergency Hospital, 400347 Cluj-Napoca, Romania; larisa_serban@yahoo.com; 7 Department of Neurosciences, “Iuliu Hațieganu” University of Medicine and Pharmacy, 400012 Cluj-Napoca, Romania; 8 Clinical CF Hospital Constanta, 1 Mai 3-5 Boulevard, 900123 Constanta, Romania; 9 Department of Neurology, Faculty of Medicine, Campus—Corp B, Ovidius University of Constanta, 1 University Alley, 900470 Constanta, Romania

**Keywords:** Parkinson’s disease, pulmonary function, impulse oscillometry, spirometry, small airway dysfunction, respiratory mechanics

## Abstract

**Background/Objectives:** Respiratory dysfunction in Parkinson’s disease (PD) is a clinically relevant but frequently underrecognized manifestation associated with functional impairment and increased risk of respiratory complications. This study compared spirometry and impulse oscillometry (IOS) in the assessment of respiratory function in PD, with particular focus on the detection of subtle or peripheral airway abnormalities. **Methods:** A prospective, single-center, cross-sectional study was conducted, including 108 participants (55 patients with PD and 53 control subjects). Pulmonary function was evaluated using standardized spirometry and IOS protocols. Group comparisons were performed using non-parametric tests, while multivariable regression analyses adjusted for potential confounding factors, including age, body mass index, smoking status, pollutant exposure, and cardiovascular comorbidities. **Results:** IOS identified a higher frequency of abnormal categorical findings compared with spirometry, including among subjects with normal spirometric values. Although dyspnea was more frequent in patients with PD in unadjusted analyses, multivariable regression demonstrated that PD was not an independent predictor of respiratory dysfunction. Pollutant exposure was significantly associated with abnormal IOS findings (*p* = 0.011). No significant differences were observed between PD and control groups regarding continuous spirometric or oscillometric parameters. Only a weak association between disease severity and FEV1 (%) was identified, whereas no significant correlations were observed for oscillometric parameters. **Conclusions:** IOS may provide complementary information regarding subtle or peripheral respiratory abnormalities in patients with PD. The findings suggest that respiratory alterations in this population are likely multifactorial and not independently determined by PD itself. Incorporating oscillometric assessment into respiratory evaluation may contribute to the identification of subtle respiratory mechanical alterations in patients with PD.

## 1. Introduction

Parkinson’s disease (PD) is a progressive neurodegenerative disorder characterized by dopaminergic neuronal loss and a broad spectrum of motor and non-motor manifestations. Beyond its classical motor phenotype, it is increasingly recognized as a multisystem disorder with significant respiratory involvement [1,2,3].

Respiratory abnormalities have been reported even in early or asymptomatic stages of PD and remain frequently underrecognized in clinical practice [4,5]. These manifestations include ventilatory dysfunction, impaired airway mechanics, and reduced effectiveness of protective reflexes such as coughing and swallowing [3,5,6]. Pulmonary complications—especially aspiration pneumonia—represent a major contributor to morbidity and are among the leading causes of mortality in patients with PD [7].

Unlike primary airway diseases such as asthma or chronic obstructive pulmonary disease (COPD), respiratory dysfunction in PD is not primarily driven by airway inflammation, bronchial hyperreactivity, or structural airway remodeling [5,6,8]. Instead, respiratory involvement in PD is considered multifactorial, resulting from a complex interaction between central respiratory dysregulation and peripheral neuromuscular impairment. Neurodegenerative changes affecting brainstem structures involved in respiratory control have been linked to impaired ventilatory drive, altered respiratory rhythm, and abnormal responses to hypoxia and hypercapnia [5,9]. At the peripheral level, several motor and neuromuscular features of PD—including chest wall rigidity, bradykinesia, postural abnormalities, impaired coordination of respiratory muscles, and abnormal control of upper airway musculature—can adversely affect thoracic compliance and breathing mechanics [3,6,8]. These changes may disrupt the coordination of inspiratory and expiratory movements, impair airway clearance, and promote heterogeneous airflow distribution, thereby contributing to ineffective ventilation and subtle mechanical alterations that are not necessarily related to primary inflammatory airway disease [5,6,8].

Consequently, pulmonary involvement in PD may present through a broad spectrum of ventilatory patterns, ranging from restrictive dysfunction and upper airway obstruction to mixed or subclinical respiratory changes that can remain undetected during routine pulmonary assessment [6,8,10]. Restrictive patterns are thought to reflect reduced chest wall mobility and respiratory muscle dysfunction, whereas upper airway abnormalities may result from impaired neuromuscular control of laryngeal and pharyngeal structures [5,8]. In addition, subtle or mixed respiratory alterations may precede clinically overt pulmonary impairment and contribute to symptoms such as dyspnea, exercise intolerance, ineffective cough, and impaired airway clearance [7,10]. Furthermore, impaired cough reflex and swallowing dysfunction may compromise airway protection mechanisms, thereby increasing the risk of pulmonary complications and aspiration-related morbidity in this population [5,7].

The assessment of pulmonary function is therefore essential for the early detection and comprehensive characterization of respiratory involvement in patients with PD [11,12]. The identification of specific ventilatory patterns may improve recognition of subtle or subclinical respiratory abnormalities and provide additional insight into the functional impact of respiratory dysfunction in this population. Spirometry remains the cornerstone of pulmonary function testing, offering quantitative evaluation of airflow and ventilatory patterns while enabling identification of obstructive and restrictive abnormalities [8,9,10]. However, conventional spirometric measurements are effort-dependent and predominantly reflect large airway function, which may limit their sensitivity for detecting subtle alterations in peripheral airway mechanics or early respiratory impairment [13,14].

In contrast, impulse oscillometry (IOS) enables non-invasive assessment of respiratory impedance during tidal breathing and provides detailed information regarding resistance and reactance across different airway regions, making it particularly sensitive to frequency-dependent alterations in peripheral airway mechanics that may not be identified by conventional spirometric indices [14,15]. Although PD is primarily considered a neurodegenerative and neuromuscular disorder, impaired respiratory muscle coordination, abnormal upper airway control, and altered chest wall mechanics may secondarily influence peripheral airway dynamics and respiratory impedance measurements. Consequently, IOS may represent a valuable complementary tool for identifying subtle or subclinical respiratory mechanical abnormalities in patients with PD, particularly in those with preserved or only mildly altered spirometric findings.

Previous studies investigating the relationship between IOS and spirometry in PD have demonstrated only partial agreement between the two techniques, with IOS identifying additional respiratory mechanical abnormalities not detected by conventional spirometric indices in some patients with normal or minimally altered spirometric values [15,16,17]. Nevertheless, the clinical significance of these oscillometric findings and their relationship with PD-related respiratory dysfunction remain insufficiently understood.

Taken together, these considerations support the need for a direct comparison between spirometry and IOS to better characterize pulmonary dysfunction in PD and to identify subclinical abnormalities that may remain undetected using conventional methods. Accordingly, the present study aims to comparatively assess pulmonary function using spirometry and IOS in patients with PD, with the objective of determining whether oscillometric parameters provide additional diagnostic value in the detection of early or peripheral airway dysfunction.

## 2. Materials and Methods

### 2.1. Study Design

This study was designed as a prospective, single-center, observational cross-sectional study with consecutive enrollment, in which all assessments were performed at a single time point. It was conducted at a tertiary care pneumology hospital specializing in respiratory medicine from May 2025 to March 2026. The study aimed to evaluate pulmonary function in patients with PD and to compare the performance of spirometry and IOS in detecting respiratory functional abnormalities.

The study protocol was reviewed and approved by the institutional Ethics Committee, in accordance with national and international ethical standards (Approval No. 5416/16.05.2025). Prior to enrollment, all participants were informed about the study objectives, procedures, and the voluntary nature of participation. Written informed consent was obtained from all participants before any clinical or functional assessments. Confidentiality of participant data was ensured throughout the study.

### 2.2. Study Population

A total of 108 participants were enrolled in the study, including a PD group (55 patients with PD) and a control group (53 individuals without PD or chronic respiratory disease). Patients with PD were recruited consecutively during routine neurological follow-up, while control participants were selected to achieve comparable demographic characteristics, particularly regarding age, sex, and body mass index (BMI). In addition, potential between-group demographic and clinical differences were further addressed through multivariable statistical adjustment.

The control group was selected from individuals evaluated in the same clinical setting who did not have PD, neurological disorders affecting respiratory function, or chronic respiratory disease. Potential between-group differences in demographic and clinical characteristics were subsequently addressed through statistical adjustment in multivariable regression analyses.

Patients were referred for pulmonary assessment during routine neurological follow-up visits, and referral was not based on predefined respiratory abnormalities or suspected pulmonary disease. Participants were not preselected according to respiratory symptoms, spirometric findings, or previous oscillometric abnormalities. Because the study was conducted in a tertiary pneumology center, the study population may have included patients with greater healthcare utilization or more complex clinical profiles compared with the general PD population. This potential selection bias should therefore be considered when interpreting the generalizability of the findings.

Additional clinical variables were systematically recorded, including disease stage according to the Hoehn and Yahr scale and current antiparkinsonian treatment, in order to characterize disease severity and its potential impact on respiratory function.

Inclusion criteria for the PD group were as follows:

- Age ≥ 18 years;
- Confirmed diagnosis of PD according to standard clinical criteria. The diagnosis of PD was established by experienced neurologists based on standard clinical diagnostic criteria routinely used in neurological practice. Neuroimaging investigations, including brain magnetic resonance imaging (MRI), were not systematically performed within the study protocol for diagnostic confirmation purposes, but were available in selected patients as part of routine neurological evaluation in order to exclude major structural or cerebrovascular pathology when clinically indicated. Formal neuropsychological testing was not systematically included in the study protocol. However, patients presenting significant cognitive impairment that could interfere with understanding or performance of pulmonary function tests were excluded based on clinical neurological assessment;
- Referral for pulmonary evaluation in a stable clinical condition;
- Ability to perform pulmonary function tests (spirometry and IOS);
- Provision of written informed consent.

The control group consisted of individuals without PD or other neurological disorders that could interfere with respiratory function, and without known chronic respiratory diseases, evaluated as part of routine clinical care. Inclusion criteria for the control group were:

- Age ≥ 18 years.
- Absence of PD or other neurological disorders.
- No history of chronic respiratory disease.
- Ability to perform reliable pulmonary function testing (spirometry and IOS).
- Provision of written informed consent.
- Exclusion criteria applied to both groups were as follows:.
- Known chronic respiratory diseases (such as chronic obstructive pulmonary disease, asthma, or interstitial lung disease).
- Acute respiratory infection within the previous 4 weeks.
- History of recent thoracic or abdominal surgery.
- Inability to perform reliable pulmonary function tests due to severe motor impairment, poor cooperation, or inadequate technique.
- Significant comorbidities that could interfere with respiratory function assessment (e.g., advanced cardiac disease or neuromuscular disorders affecting respiratory muscles).
- Cognitive impairment preventing adequate understanding or performance of the required tests.
- Current use of medications that could significantly influence respiratory function (e.g., sedatives or respiratory depressants).
- Refusal or inability to provide informed consent.

Demographic and clinical variables were systematically recorded for all participants, including age, sex, height, weight, body mass index (BMI), smoking status, exposure to environmental and occupational pollutants, respiratory symptoms, and relevant comorbidities. Exposure history was obtained through structured anamnesis based on patient self-report, encompassing both occupational and household exposures. An identical evaluation protocol was applied to both groups.

### 2.3. Pulmonary Function Tests

Pulmonary function was assessed in all participants using spirometry and IOS, performed under standardized conditions by trained personnel, with participants seated and wearing a nose clip.

#### 2.3.1. Spirometry

Spirometry remains a fundamental tool for respiratory assessment due to its wide availability and established prognostic value, particularly in identifying complications, the need for ventilatory support, and overall survival outcomes [18]. In this study, spirometry was performed using an MIR (Medical International Research, Rome, Italy) spirometer, in accordance with the recommendations of the European Respiratory Society (ERS) and the American Thoracic Society (ATS) [19].

Participants were instructed to perform a rapid maximal inspiration followed by an immediate, forceful, and sustained expiration into the spirometer, minimizing any pause at total lung capacity. The forced vital capacity (FVC) maneuver consisted of four sequential phases: maximal inspiration, an initial rapid and forceful expiratory effort (“blast”), continued expiration until no further air could be expelled or for a maximum of 15 s, followed by a rapid inspiration back to maximal lung volume. All maneuvers were performed under continuous operator supervision, with standardized verbal encouragement to ensure maximal effort and complete expiration. At least three acceptable and reproducible maneuvers were obtained [19].

The following parameters were recorded: FVC, forced expiratory volume in the first second (FEV1), the FEV1/FVC ratio, and maximal expiratory flow at 50% of FVC (MEF50) [19,20,21].

Given the use of a MIR spirometry system, spirometric results were interpreted based on the device’s embedded reference values and thresholds, expressed as percentages of predicted values, which served as the primary framework for data interpretation in this study [22]. Spirometric thresholds based on percentage of predicted values, as implemented by the MIR system, are presented in Table 1.

Within this framework, an obstructive pattern was defined by FEV1/FVC <70%, typically associated with reduced FEV1 (<80% predicted), while FVC may remain normal in early stages and decrease with disease progression. A restrictive pattern was defined by FVC <80% of predicted in the presence of a normal or increased FEV1/FVC ratio. A mixed ventilatory defect was considered when FEV1, FVC, and FEV1/FVC were all below their respective thresholds [22]. Ventilatory patterns based on percentage of predicted values, as implemented in the MIR system, are detailed in Table 2.

#### 2.3.2. IOS

IOS is a non-invasive technique used to assess respiratory mechanics during tidal breathing by superimposing external pressure oscillations, based on the forced oscillation technique (FOT) first described by Dubois et al. in 1956 [23]. IOS employs oscillatory signals across a range of frequencies (5–30 Hz), allowing the evaluation of different regions of the respiratory system, with lower frequencies (e.g., 5 Hz, R5) primarily reflecting peripheral airway function and higher frequencies (e.g., 20 Hz, R20) central airway mechanics [24,25].

IOS measurements were performed during quiet breathing, with participants seated, wearing a nose clip, and maintaining a tight seal around the mouthpiece. To minimize upper airway shunting, subjects were instructed to support their cheeks with their hands throughout the measurement. Each recording lasted approximately 20 s and was repeated until at least three acceptable measurements of good quality were obtained, in accordance with manufacturer recommendations and quality control criteria [26].

The method provides measurements of respiratory system impedance (Zrs), representing the combined mechanical properties of the airways, lungs, and thoracic cage, and its components: resistance (Rrs) and reactance (Xrs). Peripheral airway involvement can be inferred from the difference between resistance at low and high frequencies (R5–R20), while additional indices such as resonant frequency (Fres) and the area of reactance (AX) provide further insight into the elastic and dynamic properties of the respiratory system [20,25,26,27].

For the purposes of this study, IOS was performed using a Vyaire Medical system (Vyaire Medical, Mettawa, IL, USA), in accordance with current international recommendations. For exploratory and descriptive purposes, oscillometric findings were categorized according to the manufacturer’s reference standards and interpretation guidelines provided in the system’s technical documentation [26]. The corresponding cut-off values and oscillometric interpretation profiles used in this study are presented in Table 3.

It should be emphasized that IOS does not provide a definitive diagnosis of restrictive ventilatory dysfunction. Restrictive-like oscillometric patterns should therefore be interpreted cautiously and considered only suggestive of altered respiratory mechanics that may be compatible with restrictive physiology. Definitive confirmation of restrictive dysfunction requires lung volume assessment, particularly total lung capacity measurements, according to current ATS/ERS recommendations [20].

### 2.4. Data Analysis

Statistical analysis was conducted using IBM SPSS Statistics (version 27.0; IBM Corp., Armonk, NY, USA). Continuous variables were summarized as mean ± standard deviation (SD) when appropriate, while categorical variables were expressed as absolute frequencies and percentages.

The distribution of continuous variables was assessed using the Shapiro–Wilk test, which indicated a non-normal distribution for the analyzed parameters (*p* < 0.05). Consequently, non-parametric statistical methods were used for inferential analyses. Group comparisons for continuous variables were performed using the Mann–Whitney U test, while associations between variables were assessed using Spearman’s rank correlation coefficient.

Comparisons between the PD group and the control group were performed using the Chi-square (χ^2^) test for categorical variables, including demographic characteristics, comorbidities, smoking status, environmental exposure, and respiratory symptoms. When expected cell counts were below 5, Fisher’s exact test was applied.

The comparative diagnostic performance of IOS and spirometry in detecting respiratory abnormalities was assessed using the McNemar test for paired categorical data.

Associations between PD severity (Hoehn and Yahr stages) and respiratory functional parameters (spirometric and oscillometric indices) were evaluated using Spearman’s rank correlation coefficient (ρ), given the ordinal nature of disease staging and the non-parametric distribution of variables.

All statistical tests were two-tailed, and a *p*-value < 0.05 was considered statistically significant.

No formal correction for multiple comparisons (e.g., Bonferroni or false discovery rate adjustment) was applied. Given the exploratory nature of the study and the moderate sample size, the analyses were primarily intended to identify potential associations and generate hypotheses regarding respiratory dysfunction in PD. To reduce the risk of overinterpretation, the findings were interpreted cautiously, with greater emphasis placed on the overall consistency and clinical relevance of the observed associations rather than on isolated *p*-values alone.

## 3. Results

### 3.1. Characteristics of the Study Population

A total of 108 subjects were included in the study, comprising 55 patients with PD and 53 control subjects. The baseline characteristics of the study population are presented in Table 4.

The mean age of the overall cohort was 67.94 years, with a median of 69.50 years and a range between 49 and 86 years. Gender distribution was balanced between groups, with females representing 45.3% of the control group and 47.3% of the PD group.

Comorbidities were common across the study population. Hypertension was the most prevalent condition, affecting 58.5% of controls and 52.7% of PD patients. Cardiovascular comorbidities were more frequent in the PD group (40.0%) compared to controls (20.8%). The latter included mild forms of cardiac impairment, such as grade I heart failure and minor valvular abnormalities (e.g., mild mitral regurgitation), as well as other stable cardiovascular conditions that were not expected to interfere with pulmonary function assessment.

Metabolic diseases showed a similar distribution between groups (35.8% in controls vs. 34.5% in PD).

Neurovascular findings were identified exclusively in the PD group (25.5%), and consisted of age-related changes observed on brain MRI, including mild leukoaraiosis and nonspecific degenerative alterations. These findings were subtle and not associated with a history of stroke, transient ischemic attack, or clinically significant cerebrovascular pathology, and therefore did not represent exclusion criteria.

Oncological history was limited, with four patients presenting prior malignancies, including basal cell carcinoma (*n* = 2), one case of squamous cell carcinoma without evidence of recurrence, and one case of prostate cancer diagnosed seven years prior, with no signs of active disease at the time of evaluation. Regarding other clinical conditions, a lower prevalence was observed in the PD group (9.1%) compared to controls (20.8%).

Lifestyle factors revealed a heterogeneous smoking distribution. The proportion of current smokers was higher in the PD group (32.7%) compared to controls (26.4%), while ex-smokers were more frequent in the control group. Exposure to environmental pollutants was notably higher among PD patients (61.8%) compared to controls (41.5%).

Respiratory symptoms were highly prevalent, particularly dyspnea, which was reported in 89.1% of PD patients compared to 64.2% of controls. In contrast, cough was more frequently reported in the control group (60.4%) than in the PD group (45.5%). Chest constriction showed a similar distribution between groups (approximately 23%).

Overall, the study population was characterized by a high burden of comorbidities and respiratory symptoms, with certain clinical features—such as neurovascular disease, pollutant exposure, and dyspnea—being more prominent in patients with Parkinson’s disease.

Effect size analysis further supported the observed associations. Patients with PD had approximately 4.56 times higher odds of experiencing dyspnea compared to controls (OR = 4.56), with a moderate effect size (Cramer’s V = 0.30).

### 3.2. Intergroup Differences in Baseline Characteristics Between PD and Control Groups

Comparative analysis of baseline characteristics between the PD group and control subjects revealed a statistically significant difference in age, with PD patients being older on average (U = 1070.50, *p* = 0.017), while no significant difference was observed in body mass index between groups (U = 1397.00, *p* = 0.709), as detailed in Table 5.

Among clinical variables, dyspnea was significantly more frequent in patients with PD compared to controls (χ^2^ = 9.44, *p* = 0.002). Cardiovascular comorbidities were also significantly more prevalent in the PD group (χ^2^ = 4.71, *p* = 0.030), whereas neurovascular disease was identified exclusively in PD patients, showing a highly significant difference between groups (χ^2^ = 15.50, *p* < 0.001).

No statistically significant differences were observed between groups for hypertension (*p* = 0.547), metabolic diseases (*p* = 0.887), neoplasia (*p* = 0.970), depression (*p* = 0.247), gastrointestinal conditions (*p* = 0.963), or other comorbidities (*p* = 0.088). Similarly, smoking status did not differ significantly between groups (χ^2^ = 2.79, *p* = 0.248).

Although unadjusted analysis indicated higher odds of dyspnea among patients with PD, this association was further examined in multivariable models adjusting for potential confounders.

These baseline differences indicate that the PD and control groups were not fully comparable in all clinical characteristics. Therefore, unadjusted between-group comparisons should be interpreted cautiously, and greater emphasis was placed on multivariable analyses adjusted for relevant confounding factors.

### 3.3. Clinical Characteristics of Patients with PD

Clinical characteristics of the PD subgroup, including disease stage and treatment, are presented in Table 6.

The majority of patients were classified in Hoehn and Yahr stages 2 (49.1%) and 3 (41.8%), indicating a predominance of moderate disease severity within the study population, while early (stage 1) and advanced stages (stage 4) were less frequently represented.

This distribution may have reduced the ability to identify consistent stage-dependent respiratory changes across the analyzed parameters.

Regarding treatment, a heterogeneous therapeutic profile was observed. Dopamine agonists (pramipexole) and levodopa-based therapies, including standard levodopa and levodopa–carbidopa combinations, were the most commonly used regimens. Advanced therapies such as levodopa–carbidopa intestinal gel (LCIG) were also present in a subset of patients, reflecting variability in disease management strategies.

Overall, the PD cohort was characterized by predominantly moderate disease stages and diverse treatment approaches, factors that may contribute to variability in respiratory function without clear stage-dependent patterns.

### 3.4. Descriptive Analysis of Respiratory Parameters

Descriptive statistics of respiratory parameters stratified by study group are presented in Table 7.

Oscillometric parameters demonstrated substantial variability in both study groups, particularly for indices reflecting peripheral airway mechanics. Although mean spirometric values remained largely within normal ranges in both PD and control subjects, oscillometric indices suggested heterogeneous alterations in respiratory mechanics across the study population. Higher mean values for R5, R5–R20, Fres, and AX were observed in the PD group, suggesting a tendency toward increased peripheral airway involvement and altered respiratory impedance patterns. In contrast, spirometric parameters showed only limited between-group variation. Overall, these findings suggest heterogeneous respiratory mechanical alterations within the study population. However, given the absence of statistically significant between-group differences in continuous respiratory parameters, these observations should be interpreted cautiously and considered exploratory.

Comparative analysis of respiratory parameters between patients with PD and control subjects did not reveal statistically significant differences for either spirometric or oscillometric indices, as seen in Table 8.

Specifically, no significant differences were observed in spirometric parameters, including FEV1 (U = 1301.0, *p* = 0.336) and FVC (U = 1207.5, *p* = 0.124), indicating comparable ventilatory function between groups. Similarly, oscillometric parameters did not differ significantly between groups, including R5 (*p* = 0.632), R20 (*p* = 0.749), and R5–R20 (*p* = 0.387). Reactance-related indices such as Fres (*p* = 0.893), AX (*p* = 0.311), and X5 (*p* = 0.326) also showed no statistically significant differences.

However, complementary paired analysis demonstrated a significant discordance between spirometry and IOS, with IOS identifying a higher number of abnormal categorical findings. Although continuous oscillometric parameters did not differ significantly between groups, these discordant results suggest that IOS may provide complementary information regarding subtle respiratory mechanical alterations not detected by conventional spirometry. These findings should nevertheless be interpreted cautiously given the exploratory nature of the analysis and the absence of independent associations after multivariable adjustment.

### 3.5. Multivariable Regression Analysis

To account for potential confounding effects, multivariable regression analyses were performed including age, body mass index (BMI), smoking status, pollutant exposure, and cardiovascular comorbidities as covariates (Table 9).

In the adjusted logistic regression model, PD was not a significant predictor of dyspnea (OR = 0.48, *p* = 0.372). None of the covariates included showed statistically significant associations with dyspnea.

In contrast, pollutant exposure was identified as a significant independent predictor of abnormal IOS findings (OR = 0.06, *p* = 0.011), indicating a strong inverse association, while PD was not significantly associated.

Multivariable linear regression analyses showed no significant association between PD and respiratory parameters, including FEV1, R5–R20, and X5 (all *p* > 0.05). BMI was the only variable significantly associated with R5–R20 (β = 0.390, *p* = 0.015).

The multivariable regression analyses provided a more comprehensive evaluation of the relationship between PD and respiratory outcomes after adjustment for relevant confounding factors.

Although unadjusted analyses indicated a significantly higher prevalence of dyspnea among patients with PD, this association was no longer statistically significant after adjustment (OR = 0.48, *p* = 0.372). This finding suggests that the initially observed difference may be explained by confounding variables such as age, comorbidities, or environmental exposure rather than by PD itself.

Similarly, PD was not significantly associated with abnormal IOS findings (OR = 0.36, *p* = 0.138), indicating that oscillometric abnormalities was not independently associated with PD in this cohort when relevant covariates are taken into account.

In contrast, pollutant exposure emerged as a significant independent predictor of abnormal IOS results (OR = 0.06, *p* = 0.011), highlighting the potential impact of environmental factors on peripheral airway dysfunction.

In the linear regression models, PD showed no significant association with respiratory parameters, including FEV1, R5–R20, and X5 (all *p* > 0.05), further supporting the absence of an independent effect on respiratory function.

Among the analyzed covariates, BMI was the only variable significantly associated with R5–R20 (β = 0.390, *p* = 0.015), suggesting a potential influence of body composition on peripheral airway resistance.

Overall, these findings indicate that, after adjustment for potential confounding factors, an independent association between PD and respiratory dysfunction was not demonstrated in this cohort. Instead, respiratory alterations appear to be influenced by environmental exposures and individual clinical characteristics rather than by the disease itself.

### 3.6. Comparison of Diagnostic Performance and Discordance Between IOS and Spirometry

The diagnostic performance of IOS and spirometry was evaluated using the McNemar test for paired data, focusing on discordant cases defined as situations in which the two methods yielded different results.

Within the 2 × 2 contingency table, the number of cases in which IOS identified abnormalities while spirometry was normal (c = 48) was substantially higher than the number of inverse cases, in which spirometry detected abnormalities while IOS was normal (b = 10). This difference was statistically significant (χ^2^ (1) = 14.58, *p* < 0.001).

Overall, IOS identified abnormalities in 87 out of 108 subjects, whereas spirometry detected abnormalities in 49 subjects. The distribution of concordant and discordant cases is presented in Table 10.

These findings indicate a higher frequency of abnormal categorical findings identified by IOS compared with spirometry. The predominance of cases in which IOS identified abnormalities despite normal spirometric findings suggest that IOS may provide complementary information regarding subtle respiratory mechanical alterations not identified by conventional spirometry.

### 3.7. Association Between Disease Severity and Respiratory Parameters

The association between PD severity and respiratory function parameters was evaluated using Spearman correlation analysis within the PD subgroup (N = 55), as seen in Table 11.

A weak but statistically significant negative correlation was identified between PD stage and FEV1 (%) (ρ = −0.294, *p* = 0.029), suggesting lower expiratory airflow values in patients with more advanced disease stages. No statistically significant correlations were observed between PD stage and the analyzed oscillometric parameters, including R5, R20, R5–R20, Fres, AX, and X5 (all *p* > 0.05). Among the oscillometric indices, R5 demonstrated the strongest negative correlation with disease stage (ρ = −0.174, *p* = 0.205), followed by R20 (ρ = −0.136, *p* = 0.321), whereas Fres showed only a weak positive association (ρ = 0.091, *p* = 0.507). These findings suggest that, although spirometric airflow limitation may exhibit a modest association with disease severity, oscillometric abnormalities do not appear to follow a clear stage-dependent pattern in the present cohort.

## 4. Discussion

### 4.1. Summary of Main Findings

The present study provides a comprehensive evaluation of respiratory function in patients with PD using both spirometry and IOS, with a particular focus on subclinical respiratory abnormalities and the potential contribution of disease-related and non-disease-related factors.

One of the main findings of this study was that IOS identified a higher frequency of abnormal categorical findings compared with spirometry, including among subjects with normal spirometric values. This observation was further supported by the higher number of discordant cases characterized by abnormal IOS findings in the presence of normal spirometry. Although continuous oscillometric parameters did not differ significantly between groups, these findings suggest that IOS may provide complementary information regarding subtle respiratory mechanical alterations not captured by conventional spirometric indices.

Despite the higher prevalence of respiratory symptoms observed in patients with PD in the unadjusted analysis, multivariable regression models demonstrated that PD was not identified as an independent predictor of dyspnea or respiratory dysfunction after adjustment for potential confounders. The initial hypothesis that PD is independently associated with respiratory dysfunction was therefore not supported. Furthermore, only a weak association between disease severity and FEV1 was identified, while no significant correlations were observed for oscillometric parameters. These findings suggest that the apparent association between PD and respiratory abnormalities may be influenced by other factors, such as age, comorbidities, or environmental exposures, rather than by the disease itself.

In contrast, pollutant exposure emerged as a significant determinant of abnormal oscillometric findings, indicating a strong inverse association in the adjusted model. This highlights the potential role of environmental factors in influencing peripheral airway function and supports the hypothesis that respiratory alterations in this population may result from multifactorial mechanisms rather than a direct effect of PD alone.

Furthermore, no significant differences were identified between the PD and control groups in continuous spirometric or oscillometric parameters. Only a weak association between PD stage and FEV1 was observed, while no significant correlations were identified for oscillometric parameters. These findings suggest that respiratory impairment in PD may not follow a clear stage-dependent pattern, but rather reflects heterogeneous and potentially subclinical alterations.

Taken together, the results of this study suggest that IOS may provide complementary information regarding subtle respiratory mechanical alterations not identified by conventional spirometry, particularly in the context of peripheral airway dysfunction, while the independent contribution of PD to respiratory impairment appears limited after adjustment for confounding factors.

### 4.2. Comparison with Previous Studies

The findings of the present study are consistent with previous literature indicating that respiratory dysfunction represents a relevant but heterogeneous component of PD. Several studies have reported alterations in pulmonary function among patients with PD, although the type and severity of impairment vary considerably depending on disease stage, methodology, and population characteristics [10,16,28].

In agreement with prior research, the present study found no significant differences in spirometric parameters between patients with PD and control subjects. Previous studies have similarly reported that spirometry may remain within normal limits, particularly in early or moderate stages of the disease, suggesting that conventional spirometric indices may lack sensitivity for detecting subtle or peripheral airway abnormalities [15,16]. This observation is further supported by studies demonstrating that ventilatory dysfunction in PD may be underrecognized when relying exclusively on effort-dependent techniques [9,10].

The higher detection rate of abnormalities using IOS observed in the present study is in line with previous reports suggesting that oscillometric techniques may identify additional respiratory mechanical alterations not captured by spirometry. Caldas et al. demonstrated that oscillometric parameters can detect changes in respiratory mechanics even in the presence of preserved spirometric values, highlighting their potential complementary role in the assessment of peripheral airway dysfunction [15].

Similarly, Kaminsky et al. and subsequent studies have emphasized the clinical relevance of oscillometry in detecting early or subclinical respiratory impairment, particularly in conditions involving small airway dysfunction [14]. In this context, the findings of Sampath et al. provide additional support, as they reported a discrepancy between oscillometric and spirometric measurements, indicating that these methods may yield discordant results when assessing respiratory function. Notably, their work also demonstrated that oscillometric parameters, particularly those reflecting airway resistance, may detect early functional changes across different stages of PD, further supporting the potential complementary role of IOS in respiratory assessment for identifying subtle respiratory involvement [16,29].

However, not all studies report consistent findings. For example, de Oliveira et al. described normal oscillometric parameters in patients with mild PD, while spirometry suggested early obstructive changes based on flow–volume curve analysis [17]. These discrepancies may reflect differences in study design, disease severity, sample size, and methodological approaches, including variability in IOS parameters, reference values, and interpretation criteria.

Importantly, the present study extends existing literature by demonstrating that, although respiratory symptoms such as dyspnea are more prevalent in patients with PD in unadjusted analyses, PD was not an independent predictor of respiratory dysfunction after adjustment for confounding factors. This finding contrasts with some earlier studies that have suggested a direct association between PD and pulmonary impairment, but supports the notion that respiratory dysfunction in this population may be influenced by multiple interacting factors, including age, comorbidities, and environmental exposures.

In this context, the identification of pollutant exposure as a significant determinant of abnormal oscillometric findings in the present study provides additional insight into the multifactorial nature of respiratory alterations in PD and highlights the importance of considering non-neurological contributors when interpreting pulmonary function in this population. The inverse association observed may reflect residual confounding or limitations in exposure assessment.

Taken together, these findings suggest that while previous studies have established the presence of respiratory dysfunction in PD, the present results refine this understanding by indicating that such alterations may not be directly attributable to the disease itself, but rather reflect a complex interplay between disease-related and external factors, with impulse oscillometry potentially providing complementary information regarding subtle respiratory mechanical alterations.

### 4.3. Pathophysiological Considerations

From a pathophysiological perspective, the findings of the present study may be interpreted within the framework of complex interactions between central and peripheral regulatory mechanisms. Current evidence suggests that respiratory dysfunction in PD arises from a combination of these mechanisms. Neurodegenerative processes involving brainstem structures responsible for respiratory control may impair ventilatory regulation, alter respiratory rhythm, and affect responses to hypoxia and hypercapnia [5,9].

Peripheral factors further contribute to respiratory impairment. Rigidity, bradykinesia, and postural abnormalities may reduce chest wall compliance and impair respiratory muscle coordination, leading to altered breathing patterns and decreased ventilatory efficiency. In addition, upper airway dysfunction and impaired neuromuscular control may contribute to both obstructive and restrictive respiratory patterns [8,30].

Previous studies have suggested that the COVID-19 pandemic may have influenced the recognition and clinical evaluation of respiratory dysfunction in patients with chronic neurological disorders, including PD, particularly through reduced healthcare accessibility and delayed assessment of non-acute symptoms [31,32,33]. In addition, emerging evidence has explored potential interactions between SARS-CoV-2-related inflammatory mechanisms and neurodegenerative pathways involved in PD pathophysiology, although these aspects were not specifically investigated in the present study [34,35,36].

At the same time, the variability of respiratory manifestations observed in PD suggests that additional individual factors, such as comorbidities, environmental exposures, and differences in physiological reserve, may further influence functional outcomes. This heterogeneity supports the concept that respiratory involvement does not follow a uniform pattern, but rather reflects the cumulative effect of multiple interacting mechanisms [7,10,29].

Taken together, these findings support the interpretation that respiratory dysfunction in PD is multifactorial and non-linear, which may partly explain the absence of clear associations observed in the present study.

### 4.4. Lower Limit of Normal (Lln) Versus Percentage of Predicted Values in Spirometry

Traditionally, spirometric interpretation has relied on percentage of predicted values, with normal lung function often defined within the range of 80–120% of predicted. However, this approach does not adequately account for age-related variability and may lead to misclassification, particularly in younger and elderly populations. For this reason, current international guidelines recommend the use of the lower limit of normal (LLN), defined as the 5th percentile of a healthy reference population (z-score = −1.64), as a more physiologically appropriate threshold for identifying abnormal values [20,37,38,39].

In the present study, spirometric data were interpreted using percentage of predicted values due to the reference framework embedded in the MIR system used in this study. Although this approach remains commonly used in clinical practice, the use of LLN-based interpretation might have provided a more physiologically refined classification, particularly in older individuals.

From a physiological perspective, airflow obstruction is best defined by a reduction in the FEV1/FVC ratio below the LLN. Although the Global Initiative for Chronic Obstructive Lung Disease (GOLD) recommends a fixed threshold of FEV1/FVC < 0.70, this criterion may result in underdiagnosis in younger individuals and overdiagnosis in older populations due to age-related changes in lung function [40].

In contrast, restrictive patterns are suggested by concomitant reductions in FEV1 and FVC with a preserved or increased FEV1/FVC ratio, although definitive confirmation requires lung volume measurements demonstrating a total lung capacity below the LLN [20,21].

Importantly, in the context of the present study, spirometric parameters were largely within normal ranges across both groups, regardless of the interpretative approach used. This observation supports the notion that conventional spirometry—whether interpreted using percentage-based or LLN-based criteria—may have limited sensitivity for detecting early or peripheral airway dysfunction in patients with PD.

Taken together, these findings suggest that while LLN-based interpretation represents the current gold standard, the choice of interpretative framework did not substantially influence the identification of ventilatory patterns in this study. Instead, the observed discrepancies between spirometry and IOS are more likely attributable to the intrinsic limitations of spirometry in detecting small airway dysfunction rather than to differences in interpretative criteria.

### 4.5. Limitations of the Study

The present study has several limitations that should be considered when interpreting the findings. First, its single-center cross-sectional design limits the generalizability of the results and precludes any inference regarding causality or temporal relationships between PD and respiratory dysfunction. Second, although the overall sample size was adequate for exploratory comparative analyses, the relatively small number of participants may have reduced the statistical power to detect subtle group differences or weak associations, particularly in multivariable models.

Third, despite adjustment for relevant confounding factors, including age, body mass index, smoking status, pollutant exposure, and cardiovascular comorbidities, residual confounding cannot be completely excluded. This is particularly important given the significant between-group differences observed in age and selected comorbidities. In addition, some potentially relevant variables, such as the severity and duration of pollutant exposure, detailed cardiovascular status, medication effects, and physical activity level, were not quantified in greater detail and therefore could not be fully incorporated into the analyses.

Another limitation relates to the clinical status of patients with PD at the time of evaluation. All patients were assessed in the “ON” medication state, which may have influenced respiratory performance through improved motor function and coordination. It is possible that respiratory abnormalities would be more pronounced in the “OFF” state, where bradykinesia, rigidity, and impaired motor control are more evident.

Spirometric results were interpreted using percentage of predicted values rather than LLN-based criteria, due to the reference framework embedded in the MIR system used in this study. Although this approach remains clinically applicable and did not appear to substantially alter the interpretation of ventilatory patterns in the present cohort, the use of LLN-based thresholds might have provided a more refined classification, particularly in older participants.

Direct assessment of respiratory muscle strength, including maximal inspiratory and expiratory pressures (MIP and MEP), was not systematically feasible in all participants. These maneuvers require substantial patient effort, sustained cooperation, and adequate motor coordination, which may be challenging in individuals with clinically significant tremor, bradykinesia, or impaired motor control. In addition, because participants had already performed repeated effort-dependent spirometric maneuvers during the same evaluation session, additional respiratory muscle testing could have imposed further physical strain and potentially affected performance reliability. Consequently, MIP and MEP measurements could not be consistently obtained in all patients, limiting the ability to fully characterize the contribution of respiratory muscle dysfunction to the observed respiratory alterations.

In addition, several clinically relevant variables were not systematically available and therefore could not be fully incorporated into the analyses. These included disease duration, levodopa equivalent daily dose (LEDD), detailed neuromuscular functional assessment, and standardized evaluation of physical activity level. Nevertheless, most patients included in the study presented predominantly tremor-dominant motor manifestations and remained functionally active, being able to independently perform routine daily activities, particularly in earlier stages of the disease. Although these factors should be considered when interpreting the findings, the absence of detailed clinical and functional characterization may have limited a more comprehensive evaluation of the relationship between PD severity, motor impairment, and respiratory dysfunction.

Finally, exposure history and some clinical variables were based on structured self-report, which introduces the possibility of recall bias or misclassification.

Furthermore, restrictive-like oscillometric patterns identified by IOS cannot be interpreted as definitive restrictive ventilatory defects in the absence of direct lung volume measurements.

Despite these limitations, the study also has important strengths, including the combined use of spirometry and IOS, the inclusion of a control group, and the application of multivariable models to account for major confounding factors. These features support the relevance of the findings and provide a useful basis for future longitudinal and multicenter investigations.

### 4.6. Future Directions

Future research should aim to further clarify the relationship between PD and respiratory dysfunction through longitudinal and multicenter study designs, which would allow the evaluation of temporal changes and improve the generalizability of findings. In particular, prospective studies could help determine whether subclinical abnormalities detected by IOS precede clinically significant respiratory impairment.

In addition, future investigations should incorporate a more detailed characterization of environmental exposures, including quantitative assessment of pollutant type, duration, and intensity, in order to better understand their contribution to peripheral airway dysfunction. The integration of additional respiratory assessments, such as lung volumes, diffusion capacity, respiratory muscle strength, and sleep-related breathing evaluations, may provide a more comprehensive understanding of respiratory involvement in PD.

Moreover, the inclusion of patients across a broader spectrum of disease severity, including early and advanced stages, would allow a more accurate evaluation of potential stage-dependent effects. Mechanistic studies exploring the interaction between central neurological dysfunction and peripheral respiratory mechanics may also help elucidate the pathophysiological basis of the observed findings.

Finally, the potential clinical utility of IOS as a screening or monitoring tool in patients with PD warrants further investigation, particularly in the context of early detection and risk stratification of respiratory complications.

## 5. Conclusions

This study provides an exploratory comparative evaluation of spirometry and IOS in the assessment of respiratory function in patients with PD. Although IOS identified a higher frequency of abnormal categorical findings, no significant differences were observed between PD and control groups regarding continuous spirometric or oscillometric parameters after adjustment for confounding factors. In addition, PD was not identified as an independent predictor of respiratory dysfunction in multivariable analyses. These findings suggest that respiratory abnormalities in PD are likely multifactorial and heterogeneous, potentially influenced by environmental exposures and individual clinical characteristics.

IOS may nevertheless provide complementary information regarding subtle respiratory mechanical alterations not captured by conventional spirometry. Further longitudinal and larger-scale studies are required to clarify the clinical significance of these findings and the potential role of IOS in the respiratory assessment of patients with PD.

## Figures and Tables

**Table 1:** Spirometric Reference Values (MIR-based interpretation, % predicted) [20].

Parameter	Normal Values	Abnormal Values
FVC	≥80% predicted	<80%
FEV1	≥80% predicted	<80%
FEV1/FVC ratio	~70–80%	<70%

FVC—Forced Vital Capacity; FEV1—Forced Expiratory Volume in 1 s.

**Table 2:** Spirometric Ventilatory Patterns (MIR-based interpretation, % predicted) [20].

Pattern	FEV1	FVC	FEV1/FVC
Normal	≥80%	≥80%	≥ 70%
Obstructive pattern	↓	Normal or ↓	↓
Restrictive pattern	↓	↓	Normal or ↑
Mixed ventilatory defect	↓	↓	↓

FVC—Forced Vital Capacity; FEV1—Forced Expiratory Volume in 1 s; ↓—values below the defined threshold (% predicted or ratio); ↑—values above the normal range.

**Table 3:** IOS parameters and ventilatory patterns [24].

Pattern	R5 (% Predicted)	X5 (kPa·s·L^−1^, Predicted − X5)	R5–R20 (kPa·s·L^−1^)
Normal	<140%	<0.15 kPa·s·L^−1^	≤0.07 kPa·s·L^−1^
Central airway obstruction	>140%	<0.15 kPa·s·L^−1^	≤0.07 kPa·s·L^−1^
Peripheral airway obstruction	>140%	>0.15 kPa·s·L^−1^	>0.07 kPa·s·L^−1^
Restrictive-like oscillometric pattern	<140%	>0.15 kPa·s·L^−1^	≤0.07 kPa·s·L^−1^

R5—total airway resistance at 5 Hz (% predicted); R20—central airway resistance at 20 Hz; R5–R20—peripheral airway resistance index; X5—reactance at 5 Hz.

**Table 4:** Baseline characteristics of the study population.

Variable	Control (*n* = 53)	PD (*n* = 55)	Total (*n* = 108)
Age, mean ± SD	65.91 ± 7.96	69.89 ± 7.79	67.94 ± 8.09
Gender, *n* (%)			
Female	24 (45.3%)	26 (47.3%)	50 (46.3%)
Male	29 (54.7%)	29 (52.7%)	58 (53.7%)
Comorbidities, *n* (%)			
Hypertension (yes)	31 (58.5%)	29 (52.7%)	60 (55.6%)
Cardiovascular (yes)	11 (20.8%)	22 (40.0%)	33 (30.6%)
Metabolic diseases (yes)	19 (35.8%)	19 (34.5%)	38 (35.2%)
Neurovascular disease (yes)	0 (0%)	14 (25.5%)	14 (13.0%)
Neoplasia (yes)	2 (3.8%)	2 (3.6%)	4 (3.7%)
Depression (yes)	4 (7.5%)	8 (14.5%)	12 (11.1%)
Gastrointestinal (yes)	3 (5.7%)	3 (5.5%)	6 (5.6%)
Other conditions (yes)	11 (20.8%)	5 (9.1%)	16 (14.8%)
Smoking status, *n* (%)			
Never	22 (41.5%)	27 (49.1%)	49 (45.4%)
Ex-smoker	17 (32.1%)	10 (18.2%)	27 (25.0%)
Current smoker	14 (26.4%)	18 (32.7%)	32 (29.6%)
Pollutant exposure (yes)	22 (41.5%)	34 (61.8%)	56 (51.9%)
Symptoms, *n* (%)			
Dyspnea (yes)	34 (64.2%)	49 (89.1%)	83 (76.9%)
Cough (yes)	32 (60.4%)	25 (45.5%)	57 (52.8%)

**Table 5:** Comparison of baseline characteristics between PD and control groups.

Variable	Control (*n* = 53)	PD (*n* = 55)	Test Statistic	*p*
Age (years), mean ± SD	65.91 ± 7.96	69.89 ± 7.79	U = 1070.50	0.017
BMI (kg/m^2^), mean ± SD	29.40 ± 4.59	29.00 ± 3.79	U = 1397.00	0.709
Dyspnea (yes), *n* (%)	34 (64.2%)	49 (89.1%)	χ^2^ = 9.44	0.002
Hypertension (yes), *n* (%)	31 (58.5%)	29 (52.7%)	χ^2^ = 0.36	0.547
Cardiovascular (yes), *n* (%)	11 (20.8%)	22 (40.0%)	χ^2^ = 4.71	0.030
Metabolic diseases (yes), *n* (%)	19 (35.8%)	19 (34.5%)	χ^2^ = 0.02	0.887
Neurovascular disease (yes), *n* (%)	0 (0%)	14 (25.5%)	χ^2^ = 15.50	<0.001
Neoplasia (yes), *n* (%)	2 (3.8%)	2 (3.6%)	χ^2^ = 0.00	0.970
Depression (yes), *n* (%)	4 (7.5%)	8 (14.5%)	χ^2^ = 1.34	0.247
Gastrointestinal (yes), *n* (%)	3 (5.7%)	3 (5.5%)	χ^2^ = 0.00	0.963
Other conditions (yes), *n* (%)	11 (20.8%)	5 (9.1%)	χ^2^ = 2.91	0.088
Smoking status	—	—	χ^2^ = 2.79	0.248

Note: Continuous variables were compared using the Mann–Whitney U test, while categorical variables were analyzed using the Chi-square (χ^2^) test.

**Table 6:** Disease stage and treatment of PD population.

Variable	PD Group (*n* = 55)
PD stage, *n* (%)	
Stage 1	3 (5.5%)
Stage 2	27 (49.1%)
Stage 3	23 (41.8%)
Stage 4	2 (3.6%)
Treatment, *n* (%)	
Levodopa	10 (18.2%)
Levodopa–carbidopa intestinal gel (LCIG)	18 (32.7%)
Pramipexole	22 (40.0%)
Rasagiline	9 (16.4%)
Trihexyphenidyl	3 (5.5%)
Levodopa–carbidopa	14 (25.5%)
Selegiline	3 (5.5%)

**Table 7:** Descriptive statistics of respiratory parameters by study group.

Variable	HC (*n* = 53) Mean ± SD	PD (*n* = 55) Mean ± SD
FVC (%)	99.83 ± 21.39	95.56 ± 28.76
FEV1 (%)	92.44 ± 24.93	87.11 ± 25.90
R5 (%)	101.64 ± 40.03	115.56 ± 70.32
R20 (%)	96.81 ± 64.52	86.71 ± 33.47
R5–R20 (kPa/(L/s))	0.079 ± 0.081	0.144 ± 0.218
Fres (1/s)	16.27 ± 5.55	18.40 ± 8.78
AX (kPa/L)	0.77 ± 0.98	1.49 ± 2.38
X5 (%)	−0.053 ± 0.040	−0.059 ± 0.070

**Table 8:** Comparison of respiratory parameters between PD and control groups.

Variable	U	Z	*p*
FEV1 (%)	1301.0	−0.962	0.336
FVC (%)	1207.5	−1.537	0.124
R5 (%)	1379.5	−0.479	0.632
R20 (%)	1405.5	−0.320	0.749
R5–R20 (kPa/(L/s))	1317.0	−0.865	0.387
Fres (1/s)	1442.0	−0.134	0.893
AX	1292.5	−1.014	0.311
X5	1431.0	−0.982	0.326

**Table 9:** Multivariable regression analyses of respiratory outcomes. Adjusted for age, BMI, smoking status, pollutant exposure, and cardiovascular comorbidities.

Outcome	Predictor	OR (95% CI)/B (β)	*p*
Dyspnea	PD vs. Control	0.48 (0.10–2.41)	0.372
	Age	0.96 (0.83–1.11)	0.602
	BMI	1.16 (0.84–1.58)	0.369
	Smoking	—	0.274
	Pollutant exposure	1.05 (0.13–8.61)	0.965
	Cardiovascular disease	0.74 (0.09–6.05)	0.775
Abnormal IOS	PD vs. Control	0.36 (0.10–1.39)	0.138
	Age	0.97 (0.86–1.09)	0.615
	BMI	1.31 (0.99–1.74)	0.062
	Smoking	—	0.478
	Pollutant exposure	0.06 (0.007–0.52)	0.011 *
	Cardiovascular disease	1.78 (0.28–11.22)	0.541
FEV1 (%)	PD vs. Control	5.864 (0.149)	0.304
	Age	−0.118 (−0.035)	0.808
	BMI	−1.325 (−0.194)	0.227
R5–R20 (kPa/(L/s))	PD vs. Control	−0.031 (−0.093)	0.508
	BMI	0.022 (0.390)	0.015 *
X5	PD vs. Control	21.188 (0.033)	0.815

Note. OR = odds ratio; CI = confidence interval; B = unstandardized coefficient; β = standardized coefficient. * *p* < 0.05. Smoking was included as a categorical variable, and the reported *p*-values represent the overall effect in the model.

**Table 10:** Comparison of respiratory abnormality detection between IOS and spirometry.

IOS	Spirometry Abnormal	Spirometry Normal	Total
Normal	10	11	21
Abnormal	39	48	87
Total	49	59	108

Note. Discordant cases include patients in whom spirometry indicated abnormalities while IOS was normal (b = 10), and patients in whom IOS detected abnormalities despite normal spirometry (c = 48). The difference between discordant pairs was statistically significant (McNemar test: χ^2^(1) = 14.58, *p* < 0.001). Sensitivity of spirometry relative to IOS was 45%, while specificity was 52%. Cohen’s kappa coefficient indicated poor agreement between the two methods (κ = −0.02).

**Table 11:** Correlation between PD stage and respiratory parameters (N = 55).

Variable	Type	ρ (Spearman)	*p*-Value
FEV1 (%)	Spirometry	−0.294	0.029
R5 (%)	IOS	−0.174	0.205
R20 (%)	IOS	−0.136	0.321
R5–R20 (kPa/(L/s))	IOS	−0.025	0.858
Fres (1/s)	IOS	0.091	0.507
AX (kPa/L)	IOS	−0.080	0.562
X5 (%)	IOS	−0.074	0.592

## Data Availability

The original contributions presented in this study are included in the article. Further inquiries can be directed to the corresponding author.
